# HAI-178 antibody-conjugated fluorescent magnetic nanoparticles for targeted imaging and simultaneous therapy of gastric cancer

**DOI:** 10.1186/1556-276X-9-274

**Published:** 2014-05-30

**Authors:** Can Wang, Chenchen Bao, Shujing Liang, Lingxia Zhang, Hualin Fu, Yutian Wang, Kan Wang, Chao Li, Min Deng, Qiande Liao, Jian Ni, Daxiang Cui

**Affiliations:** 1 Xiangya Hospital of Central South University, 87 Xiangya Road, Changsha, Hunan 410008, People's Republic of China; 2 Institute of Nano Biomedicine and Engineering, Key Laboratory for Thin Film and Microfabrication Technology of the Ministry of Education, Department of Instrument Science and Technology, Bio-X Center, Research Institute of Translation Medicine, Shanghai JiaoTong University, Dongchuan Road 800, Shanghai 200240, People's Republic of China; 3 Department of Gastroenterology, Xi’an Central Hospital, Xi’an 71004, People's Republic of China; 4 Department of Gastroenterology, Changzhen Hospital, Shanghai 20001, People's Republic of China

**Keywords:** HAI-178 antibody, Fluorescent magnetic nanoparticles, Fluorescent imaging, Magnetic resonance imaging, Gastric cancer, Nude mice model

## Abstract

The successful development of safe and highly effective nanoprobes for targeted imaging and simultaneous therapy of *in vivo* gastric cancer is a great challenge. Herein we reported for the first time that anti-α-subunit of ATP synthase antibody, HAI-178 monoclonal antibody-conjugated fluorescent magnetic nanoparticles, was successfully used for targeted imaging and simultaneous therapy of *in vivo* gastric cancer. A total of 172 specimens of gastric cancer tissues were collected, and the expression of α-subunit of ATP synthase in gastric cancer tissues was investigated by immunohistochemistry method. Fluorescent magnetic nanoparticles were prepared and conjugated with HAI-178 monoclonal antibody, and the resultant HAI-178 antibody-conjugated fluorescent magnetic nanoparticles (HAI-178-FMNPs) were co-incubated with gastric cancer MGC803 cells and gastric mucous GES-1 cells. Gastric cancer-bearing nude mice models were established, were injected with prepared HAI-178-FMNPs via tail vein, and were imaged by magnetic resonance imaging and small animal fluorescent imaging system. The results showed that the α-subunit of ATP synthase exhibited high expression in 94.7% of the gastric cancer tissues. The prepared HAI-178-FMNPs could target actively MGC803 cells, realized fluorescent imaging and magnetic resonance imaging of *in vivo* gastric cancer, and actively inhibited growth of gastric cancer cells. In conclusion, HAI-178 antibody-conjugated fluorescent magnetic nanoparticles have a great potential in applications such as targeted imaging and simultaneous therapy of *in vivo* early gastric cancer cells in the near future.

## Background

Gastric cancer is the second most common cancer and the third leading cause of cancer-related death in China [1-3]. It remains very difficult to cure effectively, primarily because most patients present with advanced diseases [4]. Therefore, how to recognize and track or kill early gastric cancer cells is a great challenge for early diagnosis and therapy of patients with gastric cancer.

We have tried to establish an early gastric cancer pre-warning and diagnosis system since 2005 [5,6]. We hoped to find early gastric cancer cells *in vivo* by multi-mode targeting imaging and serum biomarker detection techniques [7-12]. Our previous studies showed that subcutaneous and *in situ* gastric cancer tissues with 5 mm in diameter could be recognized and treated by using multi-functional nanoprobes such as BRCAA1-conjugated fluorescent magnetic nanoparticles [13], her2 antibody-conjugated RNase-A-associated CdTe quantum dots [14], folic acid-conjugated upper conversion nanoparticles [15,16], RGD-conjugated gold nanorods [17], ce6-conjugated carbon dots [18], ce6-conjugated Au nanoclusters (Au NCs) [19,20]. However, the clinical translation of these prepared nanoprobes still exists as a great challenge because no one kind of biomarker is specific for gastric cancer. Looking for new potential biomarker of gastric cancer and development of safe and effective nanoprobes for targeted imaging and simultaneous therapy of *in vivo* early gastric cancer have become our concerns.

Dr. Jian Ni et al. found that the α-subunit of ATP synthase exhibited over-expression in breast cancer cell lines such as MCF-7H and MCF-7 cell line, with different metastasis potentials, and also exhibited high expression in breast cancer tissues, hepatocellular carcinoma, colon cancer, and prostate cancer [21]. ATP synthase is responsible for ATP production in oxidative phosphorylation and can work in reverse as a proton-pumping ATPase [22,23]. ATP synthase expression is localized exclusively in the mitochondria where it generates most cellular ATP. However, ATP synthase components have recently been identified as cell-surface receptors for apparently unrelated ligands in the course of studies carried out on angiogenesis [24-26], lipoprotein metabolism [27], innate immunity [28-32], etc. by immunofluorescence, biochemistry, and proteomics analyses. Its molecular mechanism, function, and significance have not been clarified well.

Dr. Jian Ni's group prepared specific monoclonal antibody against the α-subunit of ATP synthase, named as HAI-178 antibody, and provided this to my group. Our primary studies showed that the α-subunit of ATP synthase also exhibited over-expression in gastric cancer cells and clinical gastric cancer tissues, with no or very low expression in normal gastric mucous tissues. Especially as one kind of self antibody which existed in human sera from patients with gastric cancer, this should be a potential biomarker with diagnosis value. In our previous work, we prepared fluorescent magnetic nanoparticles (FMNPs) composed of silicon-wrapped magnetic nanoparticles and CdTe quantum dots and used FMNPs-labeled MSC cells to realize the targeted imaging and hyperthermia therapy of *in vivo* gastric cancer [33]. We also confirmed that the prepared fluorescent magnetic nanoparticles show good biocompatibility [34].

In the present study, we fully used the advantages of FMNPs and potential gastric cancer biomarker α-subunit of ATP synthase, prepared HAI-178 monoclonal antibody-conjugated FMNPs, and investigated the feasibility of prepared nanoprobes to target *in vitro* and *in vivo* gastric cancer cells. Our results show that as-prepared nanoprobes can be used for *in vivo* dual-model imaging and therapy of *in vivo* cancer, and have great potential in applications such as dual-model imaging and simultaneous therapy of early gastric cancer in the near future.

## Methods

All animal experiments (no. SYXK2007-0025) were approved by the Institutional Animal Care and Use Committee of Shanghai Jiao Tong University.

### Expression of α-subunit of ATP synthase in gastric cancer tissues

HAI-178 monoclonal antibody was presented as a gift by Dr. Jian Ni. HAI-178 monoclonal antibody was used as first antibody to stain 172 specimens of gastric cancer and control gastric mucous tissues with immunohistochemistry method [35], which were collected from Xi’an Central Hospital, Xianya Hospital, Changzheng Hospital, and the First People's Hospital in Shanghai, and identified by pathological examination.

### Preparation and Surface functionalization of FMNPs

FMNPs were prepared according to our previous report [36-38]. Before coupling the FMNPs with the HAI-178 antibody, we first functionalized the surface functional group of FMNPs as carboxyl group. Solutions of 95 mL ethanol and 2 mL 3-aminopropyltriethoxysilane (APS) were added to form a mixed solution and allowed to react at room temperature for 24 h. The aminosilane-modified FMNPs were separated by permanent magnet and were washed with deionized water three times then redispersed the FMNPs-NH_2_ in 100 mL dimethylformamide (DMF) and added with excess succinic anhydride to form a mixed solution and react at room temperature for 24 h. The carboxyl-modified FMNPs were separated by permanent magnet again and washed with deionized water three times.

### Preparation and characterization of HAI-178 monoclonal antibody-conjugated FMNPs

We used a two-step process to obtain stable HAI-178-antibody-FMNPs conjugation. Solution of 1.5 mg FMNPs-COOH was dispersed in 2 mL pH 7 PBS buffer and was sonicated for 10 min. Then we mixed 1 mL of fresh 400 mM EDC and 100 mM NHSS in pH 6.0 MES buffer and rotated it at room temperature for 15 min. After this, the resulting solution was separated by magnetic field, and 1 mg/mL of HAI-178 monoclonal antibody was added to the above mixture and stirred in dark place for 2 h. To remove free HAI-178 antibody, the residual reaction mixture was separated by magnetic field and the solid remaining was washed with 1 mL of PBS buffer three times. Finally, 1 mL of 0.05% Tween-20/PBS was added to the HAI-178 antibody-FMNPs conjugation and the bioconjugation was stored at 4°C. When used, this HAI-178 antibody-FMNPs conjugation should be diluted with PBS/0.05% Tween-20. Then we used the Nano Drop device to quantify the coupling rate of HAI-178 antibody with FMNPs-COOH. Before the coupling reaction, we measured the total concentration of HAI-178 antibody. After the coupling reaction, we measured the HAI-178 antibody concentration in residual reaction mixture and calculated the coupling rate according the equation:

Coupling (%) = (1 − Concentration of HAI-178 antibody in residual reaction mixture/Total concentration of HAI-178 antibody) × 100. The as-prepared nanoprobes and pure FMNPs were characterized by transmission electron microscopy, photoluminescence (PL) spectrometry, and fluorescent microscopy.

### Nanoprobes for *in vitro* targeting imaging of gastric cancer cells

Gastric cancer cell line MGC803 cells with over-expression of α-subunit of ATP synthase were used as target cells, and human gastric mucous GES-1 cells without expression of α-subunit of ATP synthase was used as control. The cells were cultured and collected, then were treated with 50 μg/mL HAI-178 antibody-conjugated FMNPs nanoprobes, and cultured in a humidified 5% CO_2_-balanced air incubator at 37°C for 4 h. Meanwhile, the MGC803 and GES-1 cells were treated with FMNPs as the control group. Afterward, the cells were rinsed with PBS three times, and then the cells were fixed with 2.5% glutaraldehyde solution for 30 min. For nuclear counterstaining, MGC803 cells were incubated with 1 mM Hoechst 33258 (Invitrogen, Life Technologies, Carlsbad, CA, USA) in PBS for 5 min. The cells were observed and imaged using fluorescence microscope (Nikon TS100-F, Nikon Instruments, Shanghai, China).

### Nanoprobes for fluorescence imaging of gastric cancer-bearing nude mice

Animal experiments were performed according to Guidelines for Animal Care and Use Committee, Shanghai Jiao Tong University. Male athymic nude mice were obtained from Shanghai LAC Laboratory Animal Co. Ltd., Chinese Academy of Sciences (Shanghai, China). MGC803 cells (1 × 10^6^) were injected subcutaneously into the right anterior flank area of the male nude mice with 4 to 5 weeks of age. The tumors were allowed to grow to a diameter of approximately 5 mm. At that point, about 40 μg HAI-178 antibody-FMNPs nanoprobes was injected into the mice (*n* = 3) via the tail vein. Mice were respectively monitored in a non-invasive manner at 0.5, 1, 3, 6, and 12 h to get fluorescence images. Then, tumor and major organs were collected, were placed on black papers, and subjected to IVIS Lumina imaging system (Xenogen) with emission wavelengths of 630 nm. The fluorescence images were acquired, and the total fluorescence flux for each sample was obtained. For the control experiment, mice (*n* = 3) were injected via tail vein with 40 μg of FMNPs and subjected to optical imaging at various time points post-injection. Identical illumination settings (e.g., lamp voltage, filter, exposure time) were used in all animal imaging experiments.

### Nanoprobes for MRI and fluorescent imaging of gastric cancer-bearing nude mice

For MR imaging, gastric MGC803 cells (1 × 10^6^) were injected subcutaneously into the right anterior flank area of male nude mice (*n* = 3) with 4 to 5 weeks of age. After the tumors reached approximately 5 mm in diameter, mice were injected with the HAI-178 antibody-FMNPs nanoprobes. MR imaging was performed at 6 h post-injections on animals anesthetized with 0.4% pentobarbital, using 3.0 T field intensity by GE HDX 3.0 T MR imaging instrument (GE Healthcare, Beijing, China) equipped with GE Signal Excite 3.0 T magnetic resonance imaging (MRI) software. The imaging protocol consisted of coronal and transverse T2-weighted spin echo (SE) pulse sequences. To produce T2 maps, the following imaging parameters were used: TR/TE = 1,000/10, 20, 30, 40, 40, 50, 60, 70, 80 ms; FOV = 8.0 cm; NEX = 1; slice thickness = 2.0 mm; number of excitations = 2. MR imaging was performed on the mice (*n* = 3) model with gastric tumor, and injected FMNPs without labeling HAI-178 antibody were used for the negative control. Then, the mice models with gastric cancer were injected with 40 μg HAI-178–FMNPs via the tail vein and imaged by small animal imaging system at 6 h post-injection [13].

### Nanoprobes for integrated therapy of *in vivo* gastric cancer

Gastric cancer-bearing mice were randomly divided into four groups: test group 1 (10 mice) (200 μg of HAI-178-FMNPs plus external alternating magnetic field with 63 kHz and 7 kA/m for 4 min), test group 2 (10 mice) (200 μg of FMNPs plus external alternating magnetic field with 63 kHz and 7 kA/m for 4 min), test group 3 (10 mice) (100 μg of HAI-178 antibody), and blank control (5 mice) (saline). Every 2 days, the mice sizes were measured up to 14 days, then the mice were sacrificed.

### Effects of HAI-178-FMNPs on important organs

The mice in test group were sacrificed after *in vivo* imaging. For histological evaluation, excised important organs from the heart, liver, spleen, lung, and kidney were frozen and embedded by medium at −20°C, were sectioned into 8-μm slices, were stained by hematoxylin and eosin (HE) stain method, and were observed by microscopy.

### Statistical analysis

Each experiment was repeated three times in duplicate. The results were presented as mean ± SD. Statistical differences were evaluated using the *t-*test and considered significance at *P* < 0.05.

## Results and discussion

### Expression of α-subunit of ATP synthase in gastric cancer tissues

**Figure 1:**
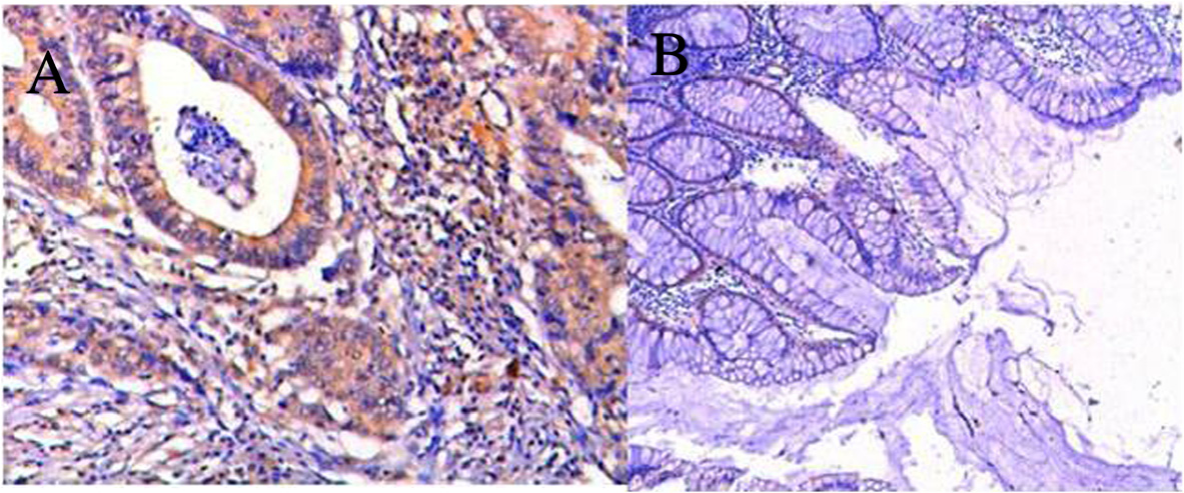
**Expression of α-subunit of ATP synthase in gastric cancer tissues and gastric mucous tissues (×50). (A)** Positive expression in gastric cancer tissues. **(B)** Negative expression in normal gastric mucous tissues.

Figure 1A showed the positive expression of α-subunit of ATP synthase in gastric cancer tissues; Figure 1B showed the negative expression of α-subunit of ATP synthase in gastric mucous tissues. We investigated the expression of α-subunit of ATP synthase in 172 specimens of gastric cancer tissues by immunohistochemistry method. As shown in Table 1, α-subunit of ATP synthase exhibited over-expression in 94.7% of the gastric cancer tissues. In no or very low expression in normal gastric mucous tissues, there existed a statistical difference between two groups (*P* < 0.01). We also observed that the expression of α-subunit of ATP synthase is not associated with patient's age (*P* > 0.05) and positive lymph node and invasion (*P* > 0.05). However, it is positively associated with the size of tumor (*P* < 0.05), pathological grade (*P* < 0.05), and TNM stage (*P* < 0.05). This result highly suggests that α-subunit of ATP synthase may be a potential biomarker for most gastric cancer patients and may be very valuable for diagnosis and therapy of clinical gastric cancer patients.

**Table 1:** Clinicopathological data and ATP synthase α-subunit expression in 172 gastric cancers

	**Description**	**α-ATP synthase expression**			**Total**	***P *** **value**
		**Negative**	**Moderate**	**Strong**		
Age	<50	5 (6.8%)	31 (42.4%)	37 (50.6%)	73 (100%)	Not significant
	≥50	4 (4.0%)	32 (32.3%)	63 (63.6%)	99 (100%)	
Size	<2 cm	2 (11.7%)	11 (64.7%)	4 (23.5%)	17 (100%)	*P* < 0.05
	≥2 cm	2 (3.4%)	22 (37.9%)	31 (53.4%)	58 (100%)	
Histological grade	Well	3 (11.5%)	12 (46.1%)	11 (42.3%)	26 (100%)	*P* < 0.05
	Moderate	7 (6.0%)	43 (37.0%)	66 (56.8%)	116 (100%)	
	Poor	0 (0.0%)	3 (15.0%)	17 (85.0%)	20 (100%)	
TNM stage	I	2 (18.1%)	5 (45.4%)	4 (36.3%)	11 (100%)	*P* < 0.05
	II	2 (4.3%)	25 (54.3%)	19 (41.3%)	46 (100%)	
	III	0 (0%)	3 (23.1)	10 (76.9%)	13 (100%)	
Lymph node invasion	Positive	3 (5.6%)	19 (35.8)	31 (58.4)	53 (100%)	Not significant
	Negative	2 (4.5%)	13 (29.5%)	31 (70.4%)	44 (100%)	

### Preparation and characterization of HAI-178-FMNPs nanoprobes

**Figure 2:**
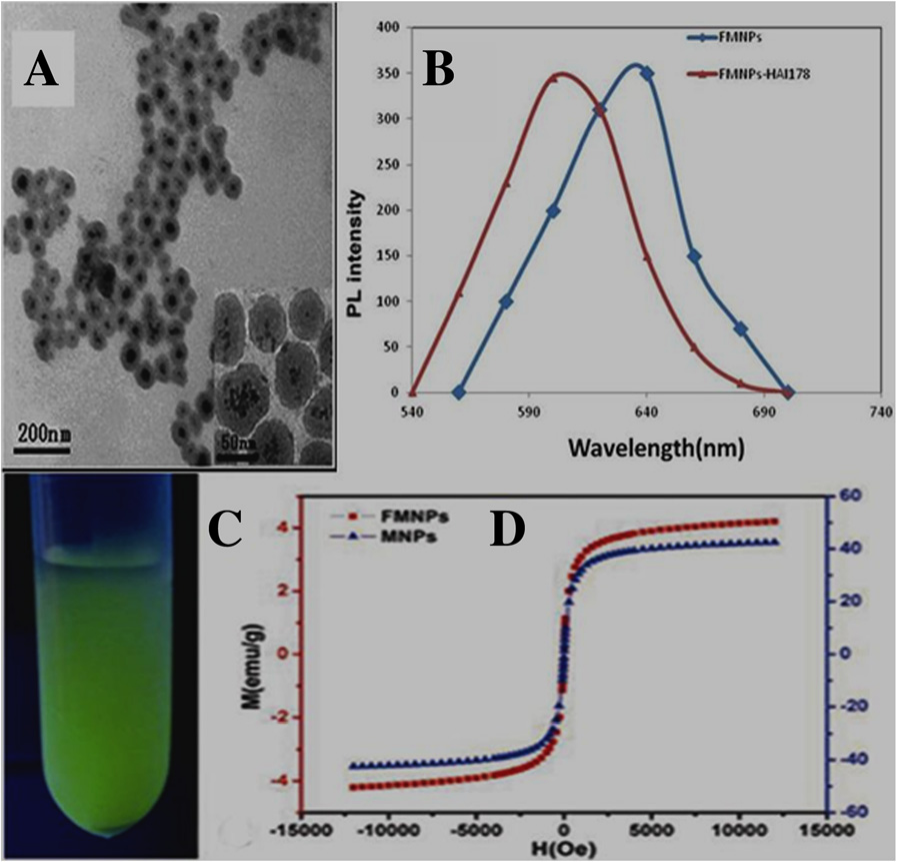
**Characterization of FMNPs and HAI-178-FMNPs. (A)** HR-TEM of FMNPs. **(B)** PL spectra of FMNPs and HAI-178-FMNPs. **(C)** Fluorescent image of prepared FMNPs. **(D)** Magnesium of FMNPs and magnetic nanoparticles

As shown in Figure 2A, prepared FMNPs were composed of silica-wrapped CdTe and magnetic nanoparticles, and their sizes were 50 nm or so in diameter. As shown in Figure 2B, after FMNPs were conjugated with HAI-178 antibody, the as-prepared nanoprobes' photoluminescence (PL) intensity was lower than that of FMNPs, exhibiting a left shift of 40 nm, which was due to the decrease in the polarization rate of the surrounding molecules, resulting in the decrease of stokes displacement and finally resulting in a blue shift in the emission spectra. Figure 2C showed that prepared FMNPs exhibited green color. Figure 2D showed that the magnesium intensity of as-prepared FMNPs and magnetic nanoparticles was 3.21 emu/g.

In the course of preparing HAI-178 antibody-FMNPs nanoprobes, we found that the surface functionalization of FMNPs was very the key to conjugate HAI-178 antibody with FMNPs via covalent bond. We observed that carboxyl groups on the surface of FMNPs conjugated with HAI-178 antibody easier than amino groups on the surface of FMNPs. In our experiment, the average coupling rate of HAI-178 antibody with FMNPs-COOH was 80.29%.

### Nanoprobes for targeting *in vitro* gastric cancer cells

**Figure 3:**
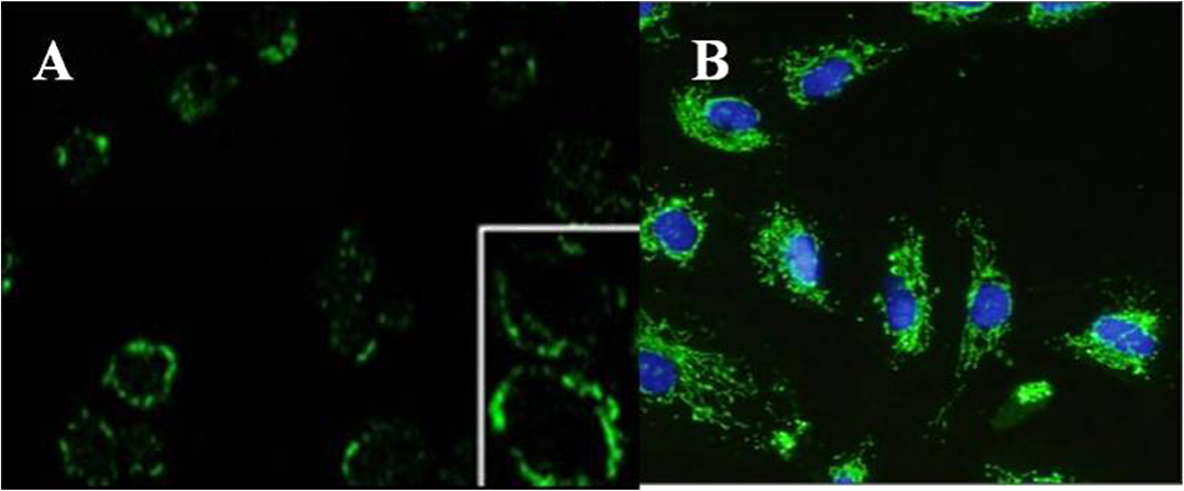
**Fluorescent microscope observation of HAI-178-FMNPs bound to surface of MGC803 cells. (A)** HAI-178-FMNPs combined to the surface of MGC803 cell membrane (×10); inset is the magnified image (×100). **(B)** HAI-178-FMNPs bound to the membrane of MGC803 cells, blue nucleus (DAPI staining) (×10).

The targeting ability of as-prepared nanoprobes *in vitro* was observed by fluorescence microscope. As shown in Figure 3A, HAI-178-conjugated FMNPs existed around MGC803 cellular membrane. HAI-178 antibody-FMNPs nanoprobes could enter into the cytoplasm of MGC803 cells after 4 h incubation with MGC803 cells, but not inside the nucleus, which highly suggests that HAI-178 antibody-conjugated FMNPs can target MGC803 cells specifically.

### Nanoprobes for fluorescent imaging of *in vivo* gastric cancer cells

**Figure 4:**
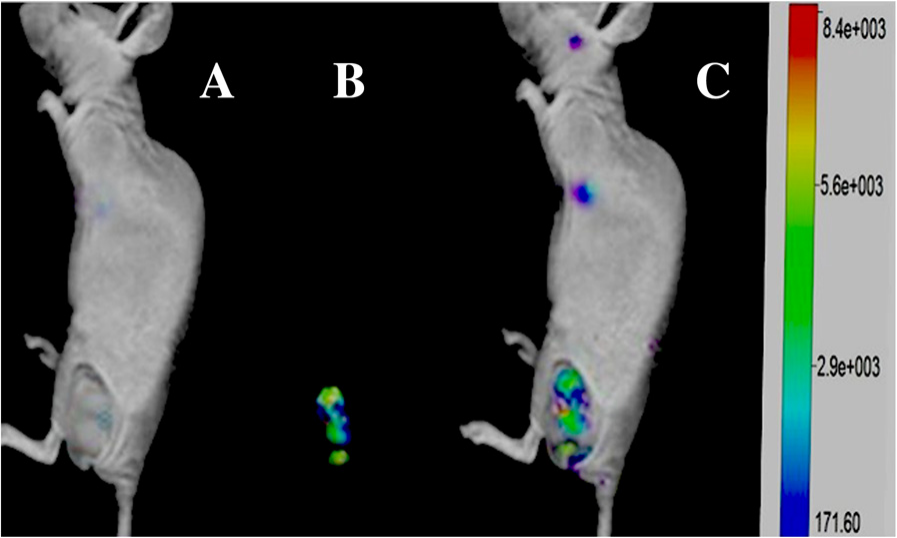
**Fluorescent imaging of gastric cancer-bearing nude mouse via tail vein injection with HAI-178-FMNPs by animal imaging system. (A)** Nude mouse loaded with gastric cancer. **(B)** Fluorescent imaging of the tumor site. **(C)** Overlay picture of gastric cancer-bearing nude mouse and fluorescent imaging of the tumor site.

To evaluate the tumor-targeting properties of HAI-178 antibody-conjugated FMNPs nanoprobes, MGC803 cells-bearing nude mice models were prepared and monitored under a non-invasive manner for 12 h by using IVIS fluorescence imaging system. Figure 4A showed the nude mouse loaded with MGC803 gastric cancer cells. Figure 4B showed the strong fluorescent signal in the tumor site of gastric cancer-bearing nude mouse at 12 h post-injection. Figure 4C showed that strong fluorescent signals only existed in the tumor site of gastric cancer-bearing nude mouse. These results indicated that the HAI-178 antibody-conjugated FMNPs were preferentially accumulated in the tumor tissues and highly suggest that prepared nanoprobes can target efficiently tumor tissues inside gastric cancer-bearing nude mice.

### Nanoprobes for MR imaging of gastric cancer-bearing nude mice

**Figure 5:**
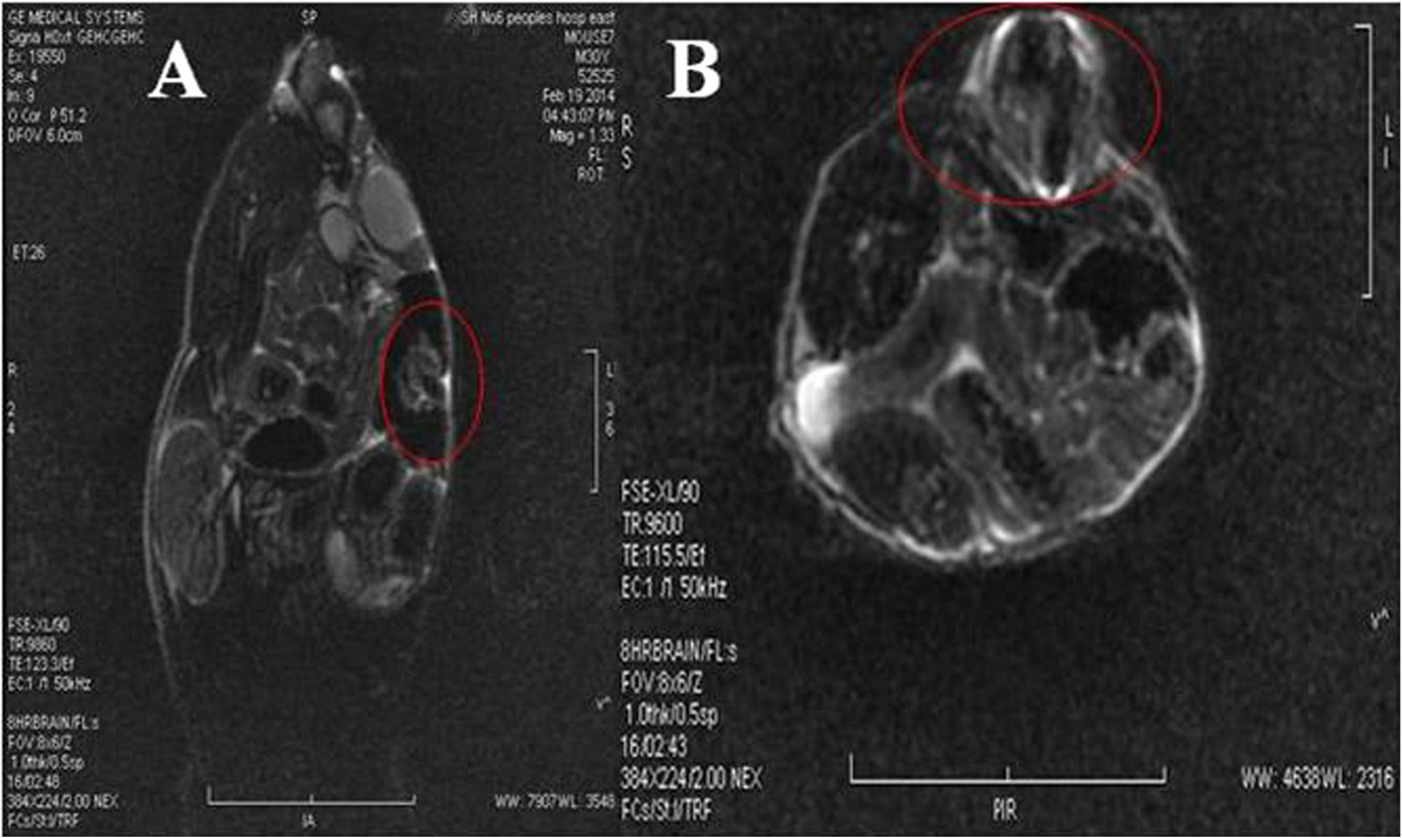
**MRI image of gastric cancer-bearing nude mouse. (A)** MRI image of nude mouse at longitudinal direction; circle shows tumor site. **(B)** MRI image of nude mice at horizontal direction; circle shows the tumor site.

*In vivo* MR imaging was performed on nude mice loaded with subcutaneous gastric cancer at 12 h post-injection. Representative images of T2 maps were shown in Figure 5. Figure 5A showed MR image of the nude mouse loaded with gastric cancer at longitudinal section, with circle showing the tumor site; a significant change in signal intensity was observed in site of tumor, indicating that there existed accumulation of the nanoprobes in the tumor site as shown in Figure 5B, showing the MR image of nude mouse at transverse direction. Our result showed that prepared nanoprobes can be used for targeted MR imaging of *in vivo* gastric cancer.

### Nanoprobes for therapy of gastric cancer-bearing nude mice

**Figure 6:**
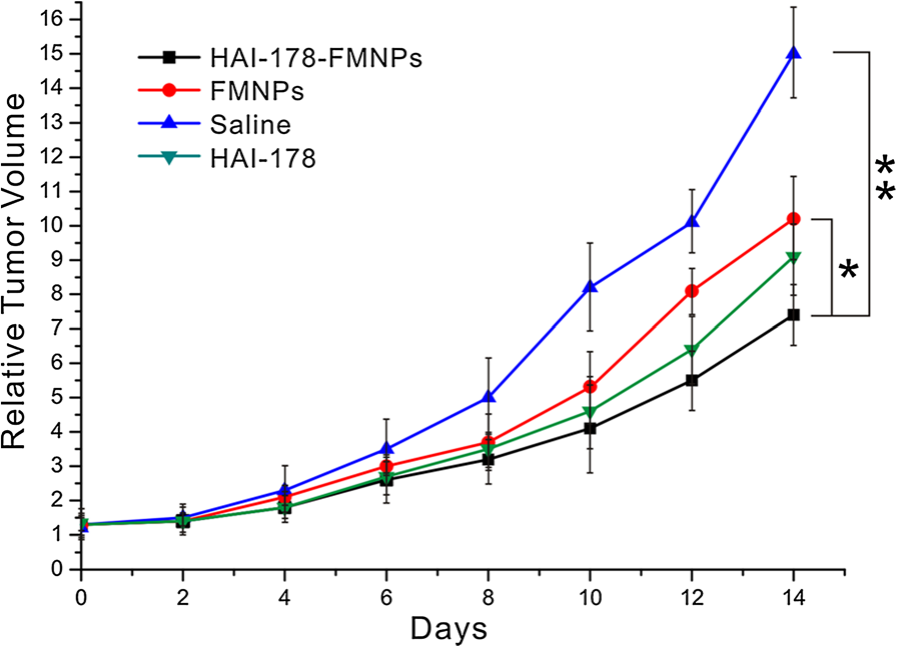
Relative tumor volume of nude mice under different treated condition.

As shown in Figure 6, the tumor tissues in control group (treated with saline) grew very quick, and the relative tumor volume became bigger and bigger as the feeding day increased. In the test group treated with FMNPs, under external alternating magnetic field with 63 kHz and 7 kA/m for 4 min, the tumor tissues in gastric cancer-bearing mice grew slower than the mice in control group. In the test group treated with HAI-178 antibody, the tumor tissues grew slower, which highly showed that HAI-178 could inhibit the growth of gastric cancer *in vivo*, similar to the inhibition of growth of breast cancer *in vivo*[26]. In test group HAI-178-FMNPs, the tumor tissues grew slowest, which highly indicate that the prepared HAI-178-FMNPs have a therapeutic function for gastric cancer *in vivo*. Compared with the control group, a statistical difference existed between two groups (*P* < 0.05). Our results showed that the prepared HAI-178-conjugated FMNPs have a therapeutic function.

### Pathological analysis of important organs

**Figure 7:**
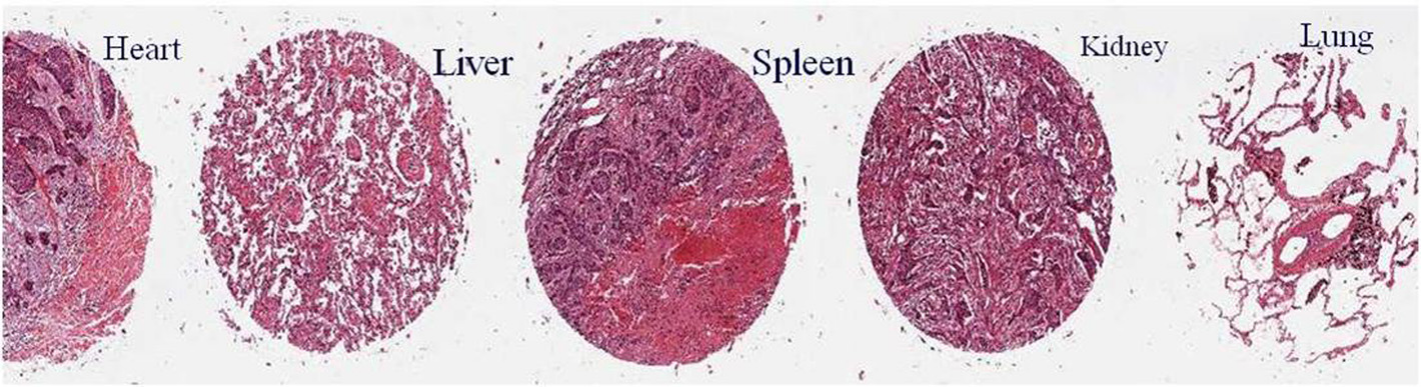
HE staining of important organs such as the heart, liver, spleen, kidney, and lung.

As shown in Figure 7, we used HE staining to check important organs including the heart, liver, spleen, lung and kidney, and no obvious damages were observed, which indirectly suggest that the prepared HAI-178-FMNPs nanoprobes did not damage important organs, showing good biocompatibility.

### Potential mechanism of targeting imaging and therapy of gastric cancer

In recent years, with the development of ‘omics,’ systems biology, and molecular imaging, a tendency of integration of multi-modality, multi-target imaging, and theranostic agents has emerged as a hotspot, a new concept called as ‘systems molecular imaging’ was suggested by Dr. Shen, which can be used to show the complexity, diversity, and *in vivo* biological behavior and the development and progress of disease in an organism qualitatively and quantitatively at a systems level. Ultimately, system molecular imaging should enable the physicians not only to diagnose tumors accurately but also to provide ‘on the spot’ treatment efficiently. It will become comprehensive research tools and technical means [39-44].

In this study, with the aim of integrating multi-mode targeted imaging and simultaneous therapy into a nanoprobe, we prepared HAI-178 antibody-conjugated FMNPs. Our previous work showed that FMNPs are very stable and have strong fluorescent signals and magnetic intensity, as well good biocompatibility. Using the strong fluorescent signals of the as-prepared nanoprobes, we successfully obtained the targeted fluorescent images of *in vivo* gastric cancer tissues in tumor-bearing nude mice, and using the strong magnetic signals of the as-prepared nanoprobes, we also successfully obtained MR images of *in vivo* gastric cancer tissues in tumor-bearing nude mice. It is confirmed that HAI-178 antibody can inhibit the growth of breast cancer cells [21]; up to date, no report is closely associated with HAI-178 antibody to inhibit growth of gastric cancer. Our results confirmed for the first time that HAI-178 antibody could be used for therapy of *in vivo* gastric cancer.

How to target *in vivo* gastric cancer cells is a key scientific problem [45]. Up to date, no specific gastric cancer biomarkers were reported. Dr. Ni et al. found that α-subunit of ATP synthase exhibited over-expression in breast cancer tissues. In our study, we confirmed that α-subunit of ATP synthase also exhibited over-expression in 94.7% of the gastric cancer specimens, which highly indicate that the α-subunit of ATP synthase may be a potential target for gastric cancer diagnosis and therapy. We also observed that the α-subunit of ATP synthase exhibited over-expression in MGC803 cells, and we used anti-α-subunit of ATP synthase antibody, that is, HAI-178 monoclonal antibody, to conjugate with florescent magnetic nanoparticles. The resultant HAI-178 antibody-conjugated FMNPs successfully realized targeted imaging and simultaneous therapy of *in vivo* gastric cancer, which highly suggests that HAI-178 antibody can target, recognize, and kill *in vivo* cancer cells, specially gastric cancer cells. Thus, the prepared nanoprobes have a great potential in applications such as targeted dual model imaging and selective therapy of early gastric cancer.

## Conclusions

We successfully prepared novel HAI-178 antibody-conjugated FMNPs nanoprobes which can be used for targeted two modal imaging of gastric cancer and have an obviously specific targeting ability toward gastric cancer tissues at 2 h of post-injection. In addition, HAI-178 antibody-conjugated FMNPs nanoprobes also exhibited inhibition of growth of gastric cancer, as first reported in this study. The as-prepared nanoprobes also can be used for hyperthermia therapy of gastric cancer under *in vitro* alternating magnetic field irradiation and have great potential in applications such as simultaneous targeted imaging and targeting therapy of clinical gastric cancer in the near future.

## Competing interests

The authors declare that they have no competing interests.

## Authors’ contributions

WC, WK, ZLX and WYT carried out clincial specimen collection. WC and LQ drafted the manuscript. BC and LS carried out the *in vitro* cell experiment. DC, LQ and NJ participated in the design of the study and performed the statistical analysis. FH and DM treated the data; LC prepared the FMNPs; BC and CC finished the animal experiment. All authors read and approved the final manuscript.

## References

[B1] Jemal A Siegel R Ward E Hao YP Xu JQ Murray T Thun MJ Cancer statistics CA Cancer J Clin 2008 9 71 96 10.3322/CA.2007.0010 18287387

[B2] Bondy M Cancer epidemiology and prevention JAMA 2009 9 1074

[B3] Okines A Verheij M Allum W Cunningham D Cervantes A Gastric cancer: ESMO clinical practice guidelines for diagnosis, treatment and follow-up Ann Oncol 2010 9 Suppl 5 v50 v54 20555102 10.1093/annonc/mdq164

[B4] Jemal A Center MM DeSantis C Ward EM Global patterns of cancer incidence and mortality rates and trends Cancer Epidemiol Biomark Prev 2010 9 8 1893 1907 10.1158/1055-9965.EPI-10-0437 20647400

[B5] Cui DX Zhang L Yan XJ Zhang LX Xu JR Guo YH Jin GQ Gomez G Li D Zhao JR Han FC Zhang J Hu JL Fan DM Gao HJ A microarray-based gastric carcinoma prewarning system World J Gastroenterol 2005 9 1273 1282 15761963 10.3748/wjg.v11.i9.1273 PMC4250672

[B6] Chen J Wang W Zhang T Ji JJ Qian QR Lu LG Fu HL Jin WL Cui DX Differential expression of phospholipase C epsilon 1 is associated with chronic atrophic gastritis and gastric cancer PLoS One 2012 9 10 e47563 10.1371/journal.pone.0047563 23077637 PMC3471869

[B7] Fu HL Ma Y Lu LG Hou P Li BJ Jin WL Cui DX TET1 exerts its tumor suppressor function by interacting with p53-EZH2 pathway in gastric cancer J Biomed Nanotechnol 2014 9 1217 1230 10.1166/jbn.2014.1861 24804542

[B8] Chen J Zhang T Feng L Zhang MQ Su HC Cui DX Synthesis of ribonuclease-A conjugated Ag_2_S quantum dots clusters via biomimetic route Mater Lett 2013 9 224 227

[B9] Cui DX Pan BF Zhang H Gao F Wu R Wang JP He R Asahi T Self-assembly of quantum dots and carbon nanotubes for ultrasensitive DNA and antigen detection Anal Chem 2008 9 7996 8001 10.1021/ac800992m 18816142

[B10] Huang P Xu C Lin J Wang C Wang X Zhang C Zhou X Guo S Cui DX Folic acid-conjugated graphene oxide loaded with photosensitizers for targeting photodynamic therapy Theranostics 2011 9 240 250 21562631 10.7150/thno/v01p0240 PMC3092447

[B11] Wang C Li ZM Liu B Liao QD Bao CC Fu HL Pan BF Jin WL Cui DX Dendrimer modified SWCNTs for high efficient delivery and intracellular imaging of survivin siRNA Nano Biomed Eng 2013 9 3 125 130

[B12] Song H He R Wang K Ruan J Bao CC Li N Ji JJ Cui DX Anti-HIF-1 alpha antibody-conjugated pluronic triblock copolymers encapsulated with Paclitaxel for tumor targeting therapy Biomaterials 2010 9 2302 2312 10.1016/j.biomaterials.2009.11.067 20004970

[B13] Wang K Ruan J Qian Q Song H Bao CC Kong YF Zhang CL Hu GH Ni J Cui DX BRCAA1 monoclonal antibody conjugated fluorescent magnetic nanoparticles for in vivo targeted magnetofluorescent imaging of gastric cancer J Nanobiotechnol 2011 9 23 10.1186/1477-3155-9-23 PMC3127991 21612621

[B14] Ruan J Song H Qian QR Li C Wang K Bao CC Cui DX HER2 monoclonal antibody conjugated RNase-A-associated CdTe quantum dots for targeted imaging and therapy of gastric cancer Biomaterials 2012 9 7093 7102 10.1016/j.biomaterials.2012.06.053 22796163

[B15] Zhou N Ni J He R Advances of upconversion nanoparticles for molecular imaging Nano Biomed Eng 2013 9 3 131 139

[B16] He M Huang P Zhang CL Hu HY Bao CC Gao G Chen F Wang C Ma JB He R Cui DX Dual phase-controlled synthesis of uniform lanthanide-doped NaGdF_4_ upconversion nanocrystals via an OA/ionic liquid two-phase system for *in vivo* dual-modality imaging Adv Funct Mater 2011 9 4470 4477 10.1002/adfm.201101040

[B17] Li ZM Huang P Zhang XJ Lin J Yang S Liu B Gao F Xi P Ren QS Cui DX RGD-conjugated dendrimer-modified gold nanorods for in vivo tumor targeting and photothermal therapy Mol Pharm 2010 9 94 104 10.1021/mp9001415 19891496

[B18] Huang P Lin J Wang XS Wang Z Zhang CL He M Wang K Chen F Li ZM Shen GX Cui DX Chen XY Light-triggered theranostics based on photosensitizer-conjugated carbon dots for simultaneous enhanced-fluorescence imaging and photodynamic therapy Adv Mater 2012 9 5104 5110 10.1002/adma.201200650 22718562 PMC3657566

[B19] Zhou ZJ Zhang CL Qian QR Ma JB He M Pan LY Gao G Fu HL Wang K Cui DX Folic acid-conjugated silica capped gold nanoclusters for targeted fluorescence/X-ray computed tomography imaging J Nanobiotechnol 2013 9 17 10.1186/1477-3155-11-17 PMC3669628 23718865

[B20] Zhang CL Zhou ZJ Qian QR Gao G Li C Feng LL Wang Q Cui DX Glutathione-capped fluorescent gold nanoclusters for dual-modal fluorescence/X-ray computed tomography imaging J Mater Chem B 2013 9 5045 5053 10.1039/c3tb20784f 32261095

[B21] Pan J Sun LC Tao YF Zhou Z Du XL Peng L Feng X Wang J Li Y-P Liu L Wu S-Y Zhang Y-L Hu S-Y Zhao W-L Zhu X-M Lou G-L Ni J ATP synthase ecto-a-subunit: a novel therapeutic target for breast cancer J Transl Med 2011 9 211 10.1186/1479-5876-9-211 22152132 PMC3254596

[B22] Muller V Cross RL The evolution of A-, F-, and V-type ATP synthases and ATPases: reversals in function and changes in the H+/ATP coupling ratio FEBS Lett 2004 9 1 1 4 15473999 10.1016/j.febslet.2004.08.065

[B23] Zhang X Niwa H Rappas M Mechanisms of ATPases–a multi-disciplinary approach Curr Protein Pept Sci 2004 9 2 89 105 10.2174/1389203043486874 15078220

[B24] Itoh H Yoshida M Yasuda R Noji H Kinosita K Resolution of distinct rotational substeps by submillisecond kinetic analysis of F1-ATPase Nature 2001 9 6831 898 904 10.1038/35073513 11309608

[B25] Wilkens S Zheng Y Zhang Z A structural model of the vacuolar ATPase from transmission electron microscopy Micron 2005 9 2 109 126 10.1016/j.micron.2004.10.002 15629643

[B26] Amzel LM Bianchet MA Leyva JA Understanding ATP synthesis: structure and mechanism of the F1-ATPase Mol Membr Biol 2003 9 1 27 33 10.1080/0968768031000066532 12745923

[B27] Rees DM Leslie AG Walker JE The structure of the membrane extrinsic region of bovine ATP synthase Proc Natl Acad Sci U S A 2009 9 21597 21601 10.1073/pnas.0910365106 19995987 PMC2789756

[B28] Champagne E Martinez LO Collet X Barbaras R Ecto-F1Fo ATP synthase/ F1 ATPase: metabolic and immunological functions Curr Opin Lipidol 2006 9 279 284 10.1097/01.mol.0000226120.27931.76 16680033

[B29] Chi SL Wahl ML Mowery YM Shan S Mukhopadhyay S Hilderbrand SC Kenan DJ Lipes BD Johnson CE Marusich MF Capaldi RA Dewhirst MW Pizzo SV Angiostatin-like activity of a monoclonal antibody to the catalytic subunit of F1F0 ATP synthase Cancer Res 2007 9 4716 4724 10.1158/0008-5472.CAN-06-1094 17510399

[B30] Moser TL Stack MS Asplin I Enghild JJ Hojrup P Everitt L Hubchak S Schnaper HW Pizzo SV Angiostatin binds ATP synthase on the surface of human endothelial cells Proc Natl Acad Sci U S A 1999 9 2811 2816 10.1073/pnas.96.6.2811 10077593 PMC15851

[B31] Talamillo A Fernandez-Moreno MA Martinez-Azorin F Bornstein B Ochoa P Garesse R Expression of the Drosophila melanogaster ATP synthase α subunit gene is regulated by a transcriptional element containing GAF and Adf-1 binding sites Eur J Biochem 2004 9 4003 4013 10.1111/j.1432-1033.2004.04336.x 15479229

[B32] Guo P Zhang C Chen C Trottier M Garver K Inter-RNA interaction of phage *φ*29 pRNA to form a hexameric complex for viral DNA transportation Mol Cell 1998 9 2 149 155 9702202 10.1016/s1097-2765(00)80124-0

[B33] Ruan J Ji JJ Song H Qian QR Wang K Wang C Cui DX Fluorescent magnetic nanoparticle-labeled mesenchymal stem cells for targeted imaging and hyperthermia therapy of in vivo gastric cancer Nanoscale Res Lett 2012 9 309 10.1186/1556-276X-7-309 22709686 PMC3441509

[B34] Ruan J Wang K Song H Xu X Ji JJ Cui DX Biocompatibility of hydrophilic silica-coated CdTe quantum dots and magnetic nanoparticles Nanoscale Res Lett 2011 9 299 10.1186/1556-276X-6-299 21711857 PMC3211365

[B35] Pan BF Cui DX Sheng Y Ozkan CG Gao F He R Li Q Xu P Huang T Dendrimer-modified magnetic nanoparticles enhance efficiency of gene delivery system Cancer Res 2007 9 8156 8163 10.1158/0008-5472.CAN-06-4762 17804728

[B36] Hu HY Yang H Huang P Cui DX Peng YQ Zhang JC Lu FY Lian J Shi DL Unique role of ionic liquid in microwave-assisted synthesis of monodisperse magnetite nanoparticles Chem Comm 2010 9 3866 3868 10.1039/b927321b 20449521

[B37] Gao G Huang P Zhang YX Wang K Qin W Cui DX Gram scale synthesis of superparamagnetic Fe_3_O_4_ nanoparticles and fluid via a facile solvothermal route Cryst Eng Comm 2011 9 1782 1785 10.1039/c0ce00584c

[B38] He R You XG Shao J Gao F Pan BF Cui DX Core/shell fluorescent magnetic silica-coated composite nanoparticles for bioconjugation Nanotechnology 2007 9 315601 10.1088/0957-4484/18/31/315601

[B39] Shen BZ Systems molecular imaging: right around the corner Nano Biomed Eng 2014 9 1 1 6

[B40] Abel B Akinsule A Andrews C Aslan K Plasmon-enhanced enzymatic reactions: a study of nanoparticle-enzyme distance- and nanoparticle loading-dependent enzymatic activity Nano Biomed Eng 2011 9 3 184 191 21949594 10.5101/nbe.v3i3.p184-191 PMC3177324

[B41] Thomas N Nanoparticles in photodynamic therapy Nano Biomed Eng 2011 9 2 137 143

[B42] Zhang JP Cui DX Nanoparticle-based optical detection of microRNA Nano Biomed Eng 2013 9 1 1 10

[B43] Sonay AY Keseroğlu K Culha M 2D gold nanoparticle structures engineered through DNA tiles for delivery, therapy Nano Biomed Eng 2012 9 1 17 22

[B44] Zhang LM Xia K Bai YY Lu ZY Tang YJ Deng Y He NY Synthesis of gold nanorods and their functionalization with bovine serum albumin for optical hyperthermia J Biomed Nanotechnol 2014 9 1440 1449 10.1166/jbn.2014.1932 25016644

[B45] Jin L Zeng X Liu M Deng Y He NY Current progress in gene delivery technology based on chemical methods and nano-carriers Theranostics 2014 9 3 240 255 10.7150/thno.6914 24505233 PMC3915088

